# Evaluation of the Spider (*Phlogiellus* genus) Phlotoxin 1 and Synthetic Variants as Antinociceptive Drug Candidates

**DOI:** 10.3390/toxins11090484

**Published:** 2019-08-22

**Authors:** Tânia C. Gonçalves, Pierre Lesport, Sarah Kuylle, Enrico Stura, Justyna Ciolek, Gilles Mourier, Denis Servent, Emmanuel Bourinet, Evelyne Benoit, Nicolas Gilles

**Affiliations:** 1 Service d’Ingénierie Moléculaire des Protéines (SIMOPRO), CEA, Université Paris-Saclay, F-91191 Gif sur Yvette, France; 2 Sanofi R&D, Integrated Drug Discovery—High Content Biology, F-94440 Vitry-sur-Seine, France; 3 Institut de Génomique Fonctionnelle (IGF), CNRS-UMR 5203, Inserm-U661, Université de Montpellier, Laboratories of Excellence—Ion Channel Science and Therapeutics, F-34094 Montpellier, France; 4 Institut des Neurosciences Paris-Saclay (Neuro-PSI), UMR CNRS/Université Paris-Sud 9197, Université Paris-Saclay, F-91198 Gif sur Yvette, France

**Keywords:** *Phlogiellus* spider, phlotoxin 1, human voltage-gated ion channel subtypes, Na_V_1.7 channel subtype, mouse model of Na_V_1.7-mediated pain

## Abstract

Over the two last decades, venom toxins have been explored as alternatives to opioids to treat chronic debilitating pain. At present, approximately 20 potential analgesic toxins, mainly from spider venoms, are known to inhibit with high affinity the Na_V_1.7 subtype of voltage-gated sodium (Na_V_) channels, the most promising genetically validated antinociceptive target identified so far. The present study aimed to consolidate the development of phlotoxin 1 (PhlTx1), a 34-amino acid and 3-disulfide bridge peptide of a *Phlogiellus* genus spider, as an antinociceptive agent by improving its affinity and selectivity for the human (h) Na_V_1.7 subtype. The synthetic homologue of PhlTx1 was generated and equilibrated between two conformers on reverse-phase liquid chromatography and exhibited potent analgesic effects in a mouse model of Na_V_1.7-mediated pain. The effects of PhlTx1 and 8 successfully synthetized alanine-substituted variants were studied (by automated whole-cell patch-clamp electrophysiology) on cell lines stably overexpressing hNa_V_ subtypes, as well as two cardiac targets, the hCa_V_1.2 and hK_V_11.1 subtypes of voltage-gated calcium (Ca_V_) and potassium (K_V_) channels, respectively. PhlTx1 and D7A-PhlTx1 were shown to inhibit hNa_V_1.1–1.3 and 1.5–1.7 subtypes at hundred nanomolar concentrations, while their affinities for hNa_V_1.4 and 1.8, hCa_V_1.2 and hK_V_11.1 subtypes were over micromolar concentrations. Despite similar analgesic effects in the mouse model of Na_V_1.7-mediated pain and selectivity profiles, the affinity of D7A-PhlTx1 for the Na_V_1.7 subtype was at least five times higher than that of the wild-type peptide. Computational modelling was performed to deduce the 3D-structure of PhlTx1 and to suggest the amino acids involved in the efficiency of the molecule. In conclusion, the present structure–activity relationship study of PhlTx1 results in a low improved affinity of the molecule for the Na_V_1.7 subtype, but without any marked change in the molecule selectivity against the other studied ion channel subtypes. Further experiments are therefore necessary before considering the development of PhlTx1 or synthetic variants as antinociceptive drug candidates.

## 1. Introduction

More than 10% of the worldwide population is complaining about pain [1]. The first order neurons involved in pain transmission from the periphery to the central nervous system are the dorsal root ganglion (DRG neurons). These neurons express a multitude of transmembrane proteins, such as G-protein-coupled receptors (GPCRs) and ion channels, which can be considered as targets for the therapeutic treatment of pain. Hence, some molecules interacting with GPCRs or ion channels have been reported to be involved in pain relief [2,3,4,5,6]. However, in developed countries, and mainly in the United States, an overconsumption of opioids is observed due to unjustified medical prescriptions, as well as acquisition of tolerance and dependence of patients to these GPCR-targeting painkillers. Finding pharmacological, chirurgical, and behavioral alternatives has thus become a priority [7,8].

In this context, venom toxins have been explored over the two last decades as an original source of innovative antinociceptive drugs targeting different ion channel subtypes. One of these toxins, the ω-conotoxin-MVIIA isolated from a cone snail which inhibits the N-type, Ca_V_2.2 subtype of voltage-gated calcium (Ca_V_) channels, was commercialized as Prialt® after FDA approval in 2004. However, the narrow spectrum of use of this molecule, its intrathecal route of administration, and associated side-effects, represent major drawbacks for the treatment of chronic debilitating pain [9]. Among voltage-gated sodium (Na_V_) channels, the Na_V_1.7 subtype was widely reported to be the most incriminating in channelopathies related to pain, although mutations in the genes encoding some of the eight other Na_V_ subtypes, identified so far, result in painful or painless diseases with strong phenotypes [10,11,12]. Hence, numerous high-throughput screenings of venom banks from various animals (spiders, scorpions, snakes, lizards, cone snails, etc.) have been performed on the Na_V_1.7 subtype, leading to the discovery of potential analgesic candidates mainly from spider venoms [13,14,15]. In fact, approximately 20 spider toxins, comprising between 26 and 35 amino acids and sharing the same inhibitor cystine knot (ICK) architectural motif, are known to inhibit with high affinity the Na_V_1.7 subtype (reviewed by [12,16]). However, the lack of target selectivity of most of these peptides greatly impairs neuromuscular or cardiac safety issues, or both, and thus compromises their clinical development as potential antinociceptive agents [17,18,19,20,21].

More than 10 years ago, and recently confirmed, a toxin identified as µ-theraphotoxin-Pspp1, also named phlotoxin 1 (PhlTx1), was purified from the venom of a *Phlogiellus* genus spider endemic to Papua New Guinea (uniprot.org/uniprot/P0DM14, [22,23,24]). PhlTx1 (4058.83 Da), characterized by a 34-amino acid sequence with 3 disulfide bridges and structured around the ICK architectural motif, was shown to belong to the Na_V_ channel spider toxin (NaSpTx) family 1. This peptide was reported to inhibit the Na_V_1.7 subtype expressed in *Xenopus* oocytes with high affinity and to produce, although with lower efficiency than morphine, analgesic effects in a mouse model of inflammatory pain [22,23,24]. However, no or only little effect of PhlTx1 was detected on nociceptive heat stimulus responses in mice, while the peptide co-administered with either the enkephalinase inhibitor thiorphan or the opioid agonist buprenorphine, at concentrations that are ineffective alone, resulted in substantial analgesia (Patent # WO 2015036734 A1, 2015, [25,26]). Moreover, application of the opioid antagonist naloxone has been reported to potentiate noxious peripheral input into the spinal cord and to dramatically reduce analgesia in Na_V_1.7-null mutant mice, as well as in a human Na_V_1.7-null mutant, suggesting that Na_V_1.7 channel blockers alone may not replicate the analgesic phenotype of null mutant humans and mice, but may be potentiated with exogenous opioids [27].

The aim of the present study was to consolidate the development of PhlTx1 as an antinociceptive agent by studying the peptide interactions with a large panel of ion channels, to ensure its neuromuscular and cardiac safety properties, and by evaluating the structure–activity relationships (SARs) of the molecule to improve its affinity and selectivity for the target of interest (i.e., the Na_V_1.7 subtype). For this purpose, in vivo experiments were first carried out to evaluate the analgesic properties of synthetic PhlTx1. Then, the effects of the peptide and alanine (Ala)-substituted variants were investigated on cell lines stably overexpressing human (h) Na_V_ subtypes, as well as two cardiac targets, hCa_V_1.2 and the hK_V_11.1 subtype of voltage-gated potassium (K_V_) channels encoded by the human *ether-a-go-go*-related gene (hERG), using automated whole-cell patch-clamp electrophysiology. Finally, computational modelling was performed to propose a 3D-structure of PhlTx1 allowing the amino acids involved in the efficiency of the molecule to be suggested.

## 2. Results

### 2.1. Chemical Synthesis and In Vitro Folding of PhlTx1

**Figure 1:**
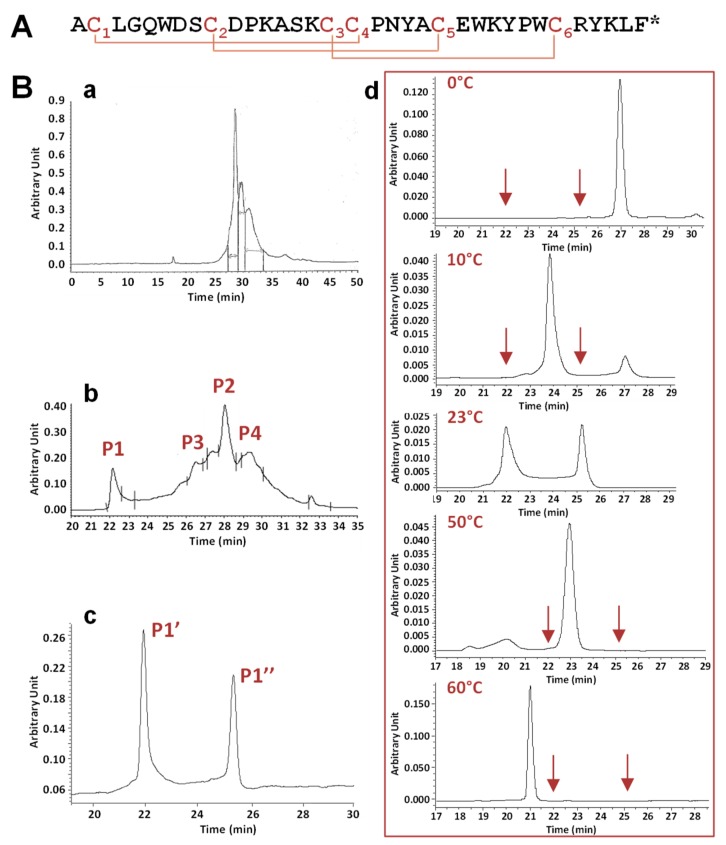
Primary structure, chemical synthesis, and in vitro folding of phlotoxin 1 (PhlTx1). (**A**) Primary structure of PhlTx1, a 34-amino acid and amidated (*) peptide including 3 disulfide bridges, as defined by homology with toxins from the Na_V_ channel spider toxin (NaSpTx) family 1, i.e., occurring according to the C1–C4, C2–C5, and C3–C6 pattern. (**B**) Chemical synthesis of PhlTx1. Analytical reversed phase high performance liquid. chromatography (RP-HPLC) profiles, detected at 214 nm, of crude peptide synthesis (**a**), crude peptide folding using the Tris-HCl buffer, the redox glutathione couple and acetonitrile (**b**), and peak P1 (**c**) detected in (**b**). Note that the presence of two peaks in (**c**) limits a non-well defined peak. (**d**) Effect of temperature on the equilibrium between the two peaks P1’ and P1’’, revealed by analytical RP-HPLC analysis of peak P1 at the indicated temperatures. The arrows indicate the elution times of peaks P1’ and P1” at 23 °C.

PhlTx1, a 34-amino acid toxin containing 6 cysteines (Figure 1A), was synthesized using the Fmoc strategy (Figure 1Ba). It was recently reported *(i)* that the random disulfide oxidation of PhlTx1 is not straightforward, *(ii)* that the directed disulfide bond formation strategy is five times more efficient than the randomized one, and *(iii)* that both strategies drive toward the same rightly folded toxin [24]. The directed disulfide bond formation strategy is usually long and requires several chemistry and purification steps. Therefore, we opted for the random disulfide oxidation strategy and tested different folding protocols according to the nature and pH of the buffer and to the redox couple used which are crucial parameters. The Tris-HCl buffer at pH 8.5 was associated with the reduced and oxidized glutathione (GSH/GSSG, 1/1) while the HEPES buffer at pH 7.5 was associated with L-cysteine/L-cystine (Cys/C–C, 1/0.1). Moreover, two chosen additives, 20% acetonitrile or glycerol, were used. The reaction was left for 48 h at 4 °C to slow down the oxidation kinetics. The best folding condition was found to be the Tris-HCl buffer and redox glutathione couple (Figure 1Bb).

Folding of the linear peptide generated a complicated profile with two main peaks, P1 and P2, with P2 being surrounded by two side-peaks, P3 and P4. Mass analysis of these four peaks revealed a similar mass corresponding to the full oxidation of the three disulfide bridges. The pharmacological activity of 1 µM of these four peaks was tested on HEK-293 cell lines stably overexpressing the hNa_V_1.7 subtype. Only peak P1 was observed to strongly inhibit the hNa_V_1.7 current, with peak P2 and peaks P3 and P4 showing moderate and no activity, respectively (data not shown). Therefore, peak P1 was further analyzed in detail by analytical RP-HPLC chromatography. The results revealed the existence of two peaks, P1’ and P1’’, of similar areas and separated by retention times of more than 3 min (Figure 1Bc). Peaks P1’ and P1’’, injected separately, generated again the two same peaks (data not shown). This demonstrates that the synthetic product equilibrated between two structural organizations, having different interaction properties with the column phase.

The influence of temperature (from 0 to 60 °C) was studied on the equilibrium between the two forms, P1’ and P1’’, of peak P1 (Figure 1Bd). Taking into account that the elution times of the different forms of PhlTx1 were inversely proportional to the temperature, decreasing the temperature from 23 to 0 °C produced a decrease of the area of peak P1’’, which finally disappeared at 0 °C, while that of peak P1’ increased. Conversely, increasing the temperature from 23 to 60 °C was favorable to peak P1’’. In agreement, Nicolas et al. [24] have recently reported the presence of two peaks generated in analytical RP-HPLC after the injection of a purified peak, unless the column was heated. The authors proposed that at least one Pro residue involved in trans/cis isomerization would be responsible for the existence of two PhlTx1 forms in equilibrium.

All of these characterizations demonstrate that the folding of PhlTx1 generates many forms visible on analytical RP-HPLC analysis. The pharmacological activity of these forms clearly indicates that the primary peak P1 was associated to the purest and the most active peptide, whatever the analytical RP-HPLC conditions were.

### 2.2. Effects of PhlTx1 on a Mouse Model of Na_V_1.7-Mediated Pain

**Figure 2:**
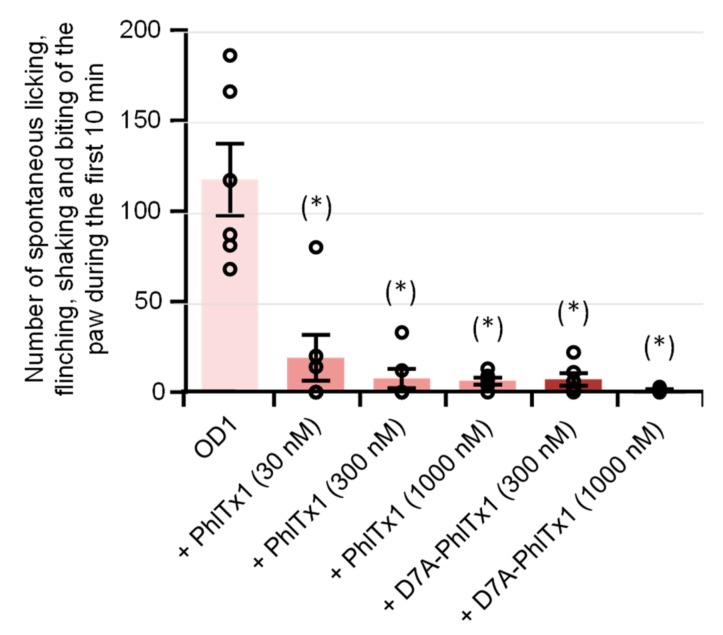
Effects of PhlTx1 and D7A-PhlTx1 on OD1-induced painful behavior of mice. Painful behavior was quantified by the number of spontaneous licking, flinching, shaking, and biting of the paw occurring during the first 10 min following intraplantar injection of PBS + 0.5% BSA + 300 nM OD1, added or not with 30, 300, or 1000 nM PhlTx1 and 300 or 1000 nM D7A-PhlTx1. Mean ± S.D. of 6 mice under each condition. (*): *P* < 0.002 when compared to OD1 (one-way ANOVA followed by post-hoc pairwise *t*-tests with Bonferroni correction).

The in vivo analgesic effects of PhlTx1 were studied in a mouse model of Na_V_1.7-mediated pain, based on intraplantar injection of toxin 1 from the *Odonthobuthus doriae* scorpion (OD1). When PBS (+0.5% BSA) containing only OD1 (300 nM) was injected into the mice, the animal painful behavior, quantified by the number of spontaneous licking, flinching, shaking, and biting of the paw, reached a maximal value between 5 and 10 min following the injection, and then disappeared progressively over 40 min. Under these conditions, the painful behavior occurring during the first 10 min following co-injection of OD1 together with PBS was 117.5 ± 18.1 (n = 6 mice). This painful behavior was markedly and significantly (*P* < 0.002) decreased by the co-injection of PBS, OD1, and 30, 300, or 1000 nM PhlTx1, i.e., 19.0 ± 11.6 (n = 6 mice), 7.5 ± 5.0 (n = 6 mice), and 6.2 ± 1.7 (n = 6 mice), respectively (Figure 2), indicating that these peptide concentrations almost completely reversed the OD1-induced pain.

### 2.3. Effects of PhlTx1 on hNa_V_1.1-1.8, hCa_V_1.2, and K_V_11.1 Channel Subtypes

**Figure 3:**
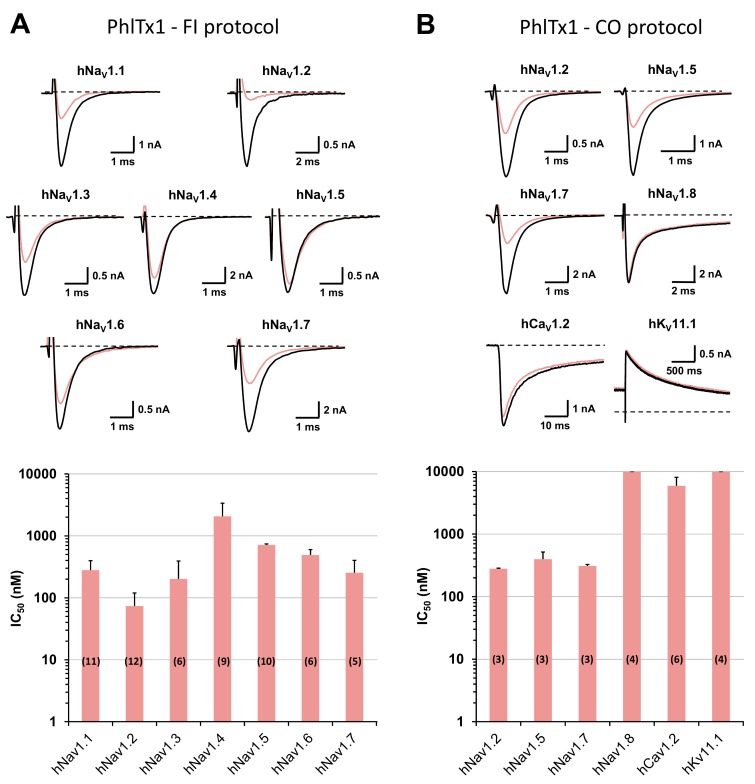
Effects of PhlTx1 on HEK-293 cells overexpressing hNa_V_ subtypes and on CHO cells overexpressing hCa_V_1.2 and hK_V_11.1 subtypes, using whole-cell automated patch-clamp. (**A**) Representative traces of sodium currents flowing through hNa_V_1.1–1.7 channel subtypes and recorded during a “fully inactivated” (FI) protocol, before (in black) and after (in pink) exposure to 350 nM PhlTx1 (upper), and histogram of IC_50_ values (in nM) obtained from the concentration–response curves of PhlTx1 effects on hNa_V_1.1–1.7 currents (lower). Each value represents the mean ± S.D. of data obtained from n different plates (numbers in parentheses) realized in triplicate. Taking into account all concentration–response curves, the mean value ± S.D. of n_H_ was 1.15 ± 0.42. (**B**) Representative traces of currents flowing through the indicated channel subtypes and recorded during a “closed-open” (CO) protocol, before (in black) and after (in pink) exposure to 350 nM PhlTx1 (upper), and histogram of IC_50_ values (in nM) obtained from the concentration–response curves of PhlTx1 effects on hNa_V_1.2, 1.5 and 1.7–1.8, hCa_V_1.2 and hK_V_11.1 currents (lower). Each value represents the mean ± S.D. of data obtained from n different plates (numbers in parentheses) realized in triplicate. Taking into account all concentration–response curves, the mean value ± S.D. of n_H_ was 1.05 ± 0.15. The recording protocols are detailed in the Materials and Methods section.

Whole-cell automated patch-clamp (QPatch HTX) experiments were performed on HEK-293 cells overexpressing hNa_V_1.1–1.8 channel subtypes, using the two electrophysiological protocols detailed in the Materials and Methods section. During the classical “closed-open” protocol, all of the hNa_V_ subtypes were maintained in a resting state before the stimulation. Conversely, during the “fully inactivated” protocol, most of the hNa_V_ subtypes were maintained in an inactivated state before the stimulation. These two different protocols allowed to test an eventual inactivated-state-dependent effect of PhlTx1 on the hNa_V_ subtypes. The results revealed, whatever the electrophysiological protocol was, that 1 μM PhlTx1 was effective to inhibit hNa_V_1.1–1.7 currents, without any marked subtype selectivity, while the hNa_V_1.8 current was unaffected (Figure 3A,B). Hence, the following increasing order of IC_50_ values was obtained from the concentration–response curves of PhlTx1 effects on currents flowing through the different channel subtypes: hNa_V_1.2 (73.7 ± 46.3 nM, n = 12 plates) ≈ hNa_V_1.3 (201.5 ± 109.8 nM, n = 6 plates) ≈ hNa_V_1.7 (254.3 ± 147.6 nM, n = 5 plates) ≈ hNa_V_1.1 (280.3 ± 117.7 nM, n = 11 plates) < hNa_V_1.6 (491.2 ± 109.0 nM, n = 6 plates) << hNa_V_1.4 (> 2.1 ± 1.3 µM, n = 9 plates) for tetrodotoxin-sensitive (TTX-S) channel subtypes, and hNa_V_1.5 (710.6 ± 32.7 nM, n = 10 plates) < hNa_V_1.8 (> 10 μM, n = 4 plates) for tetrodotoxin-resistant (TTX-R) channel subtypes. Thus, all the TTX-S hNa_V_ channel subtypes studied, with the exception of hNa_V_1.4, were sensitive to PhlTx1, as well as the TTX-R cardiac sodium channel subtype hNa_V_1.5 (although at hundred nanomolar concentrations), while the TTX-R hNa_V_1.8 subtype was resistant to the peptide (at ten micromolar concentrations). Additionally, PhlTx1 had a very low affinity for cardiac hCa_V_1.2 and hK_V_11.1 channel subtypes overexpressed in CHO cells, since 10 μM of peptide had no effect on the hK_V_11.1 current (n = 4 plates) and had only a low effect on the hCa_V_1.2 current (IC_50_ = 5.9 ± 2.1 µM, n = 6 plates; Figure 3B).

### 2.4. Structure–Activity Relationships of PhlTx1 Using an Alanine-Substituted Approach

The SAR study was carried out on PhlTx1 by replacing each amino acid of its sequence with an Ala residue which possesses a non-bulky and chemically inert methyl group, with two exceptions: the first amino acid, which was a pyroglutamic acid-modified Ala, and the five C-terminal amino acids that were removed. This Ala-substituted approach thus has the advantage to highlight the importance of each amino acid in the peptide activity.

Linear forms of all peptide variants were synthesized and purified. The experimental conditions of their folding were similar to those used to perform the folding of PhlTx1 (see Section 2.1). However, only 8 of 21 variants were obtained with adequate biophysical characteristics (Table 1). These 8 variants equilibrated between two forms, as observed for PhlTx1. The 13 other variants (L3A, Q5A, W6A, D10A, P11A, S14A, P18A, N19A, Y20A, E23A, P27A, W28A, and Delta-C-ter, i.e., PhlTx1 without its five last C-terminal residues) resulted in misfolded toxins, even after testing several different folding conditions.

**Figure 4:**
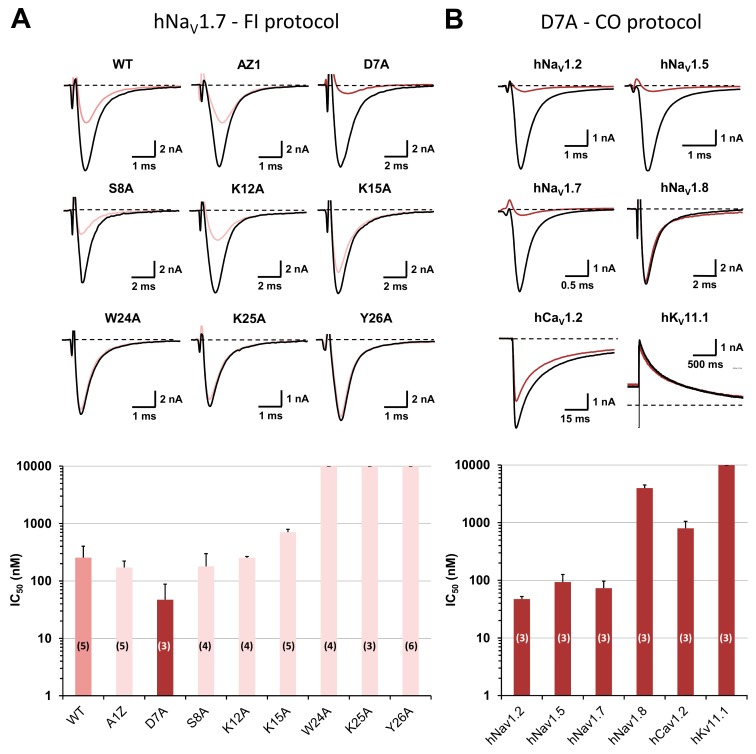
Structure–activity relationships of PhlTx1 using an alanine-substituted approach and whole-cell automated patch-clamp. (**A**) Effects of PhlTx1 wild-type (WT) and variants on HEK-293 cells overexpressing the hNa_V_1.7 subtype. Representative traces of the sodium current recorded during a “fully inactivated” (FI) protocol, before (in black) and after (in color) exposure to 350 nM PhlTx1 wild-type and the indicated variants (upper), and histogram of IC_50_ values (in nM) obtained from the concentration–response curves of peptide effects on the current (lower). Each value represents the mean ± S.D. of data obtained from n different plates (numbers in parentheses) realized in triplicate. Taking into account all concentration–response curves, the mean value ± S.D. of n_H_ was 0.99 ± 0.42. (**B**) Effects of D7A-PhlTx1 on HEK-293 cells overexpressing hNa_V_1.2, 1.5 and 1.7–1.8 subtypes and on CHO cells overexpressing hCa_V_1.2 and hK_V_11.1 subtypes. Representative traces of currents flowing through the indicated channel subtypes and recorded during a “closed-open” (CO) protocol, before (in black) and after (in brown) exposure to 350 nM D7A-PhlTx1 (upper), and histogram of IC_50_ values (in nM) obtained from the concentration–response curves of peptide effects on the currents (lower). Each value represents the mean ± S.D. of data obtained from n different plates (numbers in parentheses) realized in triplicate. Taking into account all concentration–response curves, the mean value ± S.D. of n_H_ was 1.06 ± 0.13.

The presence of Ala-substituted amino acids close to the peptide C-terminus produced a loss of variant affinity for the hNa_V_1.7 subtype, whereas little effect or even a gain of affinity of variants was detected when the substituted amino acids were closer to the peptide N-terminus (Figure 4A and Table 1). In particular, D7A-PhlTx1 had a 5.4-fold higher affinity for the hNa_V_1.7 subtype than the wild-type peptide when using the “fully inactivated” protocol [i.e., 47.0 ± 40.9 nM (n = 3 plates) and 254.3 ± 147.6 nM (n = 5 plates), respectively]. Indeed, when using the classical “closed-open” protocol, this variant gained also in affinities for hNa_V_1.7 and hNa_V_1.5 subtypes [73.0 ± 23.7 nM vs. 308.9 ± 17.0 nM (n = 3 plates), and 93.3 ± 32.9 nM vs. 395.6 ± 121.6 nM (n = 3 plates), respectively], for the hNa_V_1.2 subtype [47.3 ± 5.2 nM vs. 279.4 ± 6.1 (n = 3 plates)], and for hNa_V_1.8 and hCa_V_1.2 subtypes [4.0 ± 0.5 µM vs. > 10 µM (n = 3–4 plates), and 799.6 ± 253.4 nM vs. 5.9 ± 2.2 µM (n = 3–6 plates), respectively], while still remaining inactive on the K_V_11.1 subtype (Figure 3B and Figure 4B).

The in vivo analgesic effects of D7A-PhlTx1 were also studied on the OD1-induced painful behavior of mice. The mouse painful behavior occurring during the first 10 min was markedly and significantly (*P* < 0.0003) decreased following the co-injection of PBS, OD1 (300 nM), and 300 or 1000 nM D7A-PhlTx1, i.e., 7.0 ± 3.1 (n = 6 mice) and 1.3 ± 0.5 (n = 6 mice), respectively, compared to that following the injection of only PBS and OD1, i.e., 117.5 ± 18.1 (n = 6 mice) (Figure 2). As observed with PhlTx1, these D7A-PhlTx1 concentrations almost completely reversed the OD1-induced pain.

### 2.5. Comparison of Amino Acid Sequences and 3D-Structures of PhlTx1 with Closely Related Toxins

**Figure 5:**
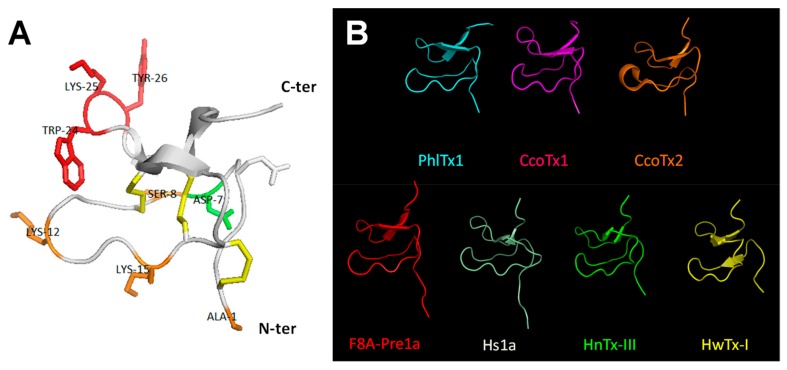
3D-Structure modelling of PhlTx1. (**A**) Ribbon representation indicating elements of secondary structure. The structure topology is composed of a double stranded antiparallel β-sheet (arrows). The three disulfide bonds are C2–C17, C9–C22, and C16–C29 (in yellow). The Ala-substituted amino acids producing a complete loss of variant affinity for the hNa_V_1.7 subtype (IC_50_ > 10 µM) are colored red (W24A, K25A and Y26A), those resulting in a less than 2.8-fold change variant affinity are colored orange (A1Z, S8A, K12A and K15A), and the one (D7A) resulting in a gain of variant affinity (more than 5-fold higher affinity) is colored green (see also Table 1). (**B**) Backbone peptide folding of PhlTx1 and toxins with PDB entries and showing the highest percentage of amino acid sequence identity with PhlTx1 (PDB entries of CcoTx1: 6BRO, CcoTx2: 6BTV, F8A-Pre1a: 5I1X, Hs1a: 2MT7, HnTx-III: 2JTB, and HwTx-I: 1QK6).

The 3D-structure of PhlTx1, containing the ICK motif in its backbone conformation expected by the amino acid sequence, was modelled by structural homology with the closest toxins from NCBI basic local alignment search tool (BLAST) (Figure 5A and Table 2). As shown in Figure 5A, the Ala-substituted amino acids producing a complete loss of variant affinity for the hNa_V_1.7 subtype are indicated in red (W24A, K25A and Y26A; IC_50_ > 10 µM, see Table 1); those resulting in a weak (less than 3-fold) modification of variant affinity are indicated in orange (A1Z, S8A, K12A and K15A; 171 nM ≤ IC_50_ ≤ 706 nM, see Table 1); and the only one resulting in a more than 5-fold higher variant affinity is indicated in green (D7A). Six of the 14 toxins extracted from NCBI BLAST had their 3D-structure defined by 2D-homonuclear ^1^H-NMR spectroscopy, which helped the computational determination of PhlTx1 3D-structure. All the 3D-structures were similar except those of F8A-Pre1a and Hs1a, which displayed the 3D-structure of peptide precursors with a long N-terminal, and those of CcoTx2 and HwTx-I pointing out three β leaflets instead of two for the other peptides (Figure 5B).

All of the toxins sharing between 45 and 76% of sequence identity with PhlTx1 were Na_V_, K_V_, and Ca_V_ channel ligands (Table 2). All of them possess three strictly conserved amino acids (G4, P11, and W28). Some of these toxins, such as Cd1a, CcoTx1, and HnTx-III, were described to target the Na_V_1.7 subtype with nanomolar affinities (IC_50_ between 2 and 232 nM, Table 2) and, thus, to have potential implications in the pain therapy field [28,29,30].

## 3. Discussion

The present study was undertaken to better characterize PhlTx1, previously described as a potential antinociceptive agent, in order to consolidate the clinical development of this peptide. The research strategy consisted of (1) the in-house chemical synthesis of PhlTx1, (2) a deep pharmacological evaluation (from in vivo to individual cells) of the synthetic peptide, (3) a SAR study carried out on PhlTx1, using an Ala-substituted approach, to improve the peptide affinity and selectivity, and (4) a computational modelling of 3D-structure to better understand the peptide effects already studied for closely related toxins.

The effects of synthetic PhlTx1 were studied on HEK-293 cells overexpressing hNa_V_1.1–1.8 subtypes and on CHO cells overexpressing hCa_V_1.2 and hK_V_11.1 subtypes, using a whole-cell automated patch-clamp technique. This study was performed *(i)* to reinforce, on human Na_V_ subtypes, the previous evaluation of the peptide selectivity profile performed on undetailed species of Na_V_ subtypes, and *(ii)* to ensure its cardiac safety properties. More than ten years ago, the effects of PhlTx1 were studied on different Na_V_ subtypes expressed with the β1 subunit in *Xenopus* oocytes [22,23]. At a concentration of 1 µM, the peptide produced greater than 90% inhibition of Na_V_1.7 current, a 35–39% inhibition of Na_V_1.4 and 1.6 currents, and less than 10% inhibition of Na_V_1.1–1.3, 1.5 and 1.8 currents, as well as of *Drosophila* Para/TipE current. The comparison of these results obtained from undetailed or *Drosophila* species of Na_V_ subtypes expressed in *Xenopus* oocytes with those we obtained from human Na_V_ subtypes stably overexpressed in mammalian cell lines is not accurate, although similar mean IC_50_ values were determined for PhlTx1 interaction with the Na_V_1.7 subtype (i.e., 260 and 254–309 nM, respectively). Therefore, this study allows the confirmation of both the peptide efficacy on the human Na_V_1.7 subtype and some of its cardiac safety properties since, in particular, the two cardiac hCa_V_1.2 and hK_V_11.1 targets were relatively saved (i.e., IC_50_ ≥ 5.9 µM) compared to the hNa_V_1.5 subtype (i.e., IC_50_ = 710.6 nM).

Compared to toxins belonging to the NaSpTx family 1, such as HnTx-I, HnTx-III, Hd1a, HnTx-IV, ProTx-III, Cm1a, GpTx-1, and HwTx-IV, previously reported in the literature to interact with the hNa_V_1.7 subtype, PhlTx1 is among the lowest potent peptides [13,14,28,29,34,37,38,39,40] (but see [21]). In addition, the peptide displayed a less than 10-fold selectivity towards the TTX-R (with the exception of hNa_V_1.8) and other TTX-S (with the exception of hNa_V_1.4) hNa_V_ subtypes (mean IC_50_ values between 74 and 711 nM). It is worth noting that relatively similar IC_50_ values were obtained from the “fully inactivated” and classical “closed-open” protocols, suggesting that no inactivated-state-dependent effect of PhlTx1 occurred on the studied hNa_V_ subtypes (see also [24]). The poor peptide selectivity towards the hNa_V_1.1 and 1.3 subtypes may be an advantage since these two subtypes have been reported to also be involved in pain pathways (see for review [12]). That towards the hNa_V_1.2 subtype, located at the level of the central nervous system, will have no important consequence taking into account the peripheral administration of PhlTx1. Therefore, the only problem of the peptide is its poor selectivity towards the hNa_V_1.5 and 1.6 subtypes since its potential use as an analgesic molecule may be associated with in vivo cardiac and neuromuscular side-effects, respectively.

The antinociceptive properties as well as the cardiac and neuromuscular side-effects of PhlTx1 were evaluated in vivo on OD1-induced painful behavior of mice. The results revealed that the peptide exhibited antinociceptive properties since intraplantar co-injection of OD1 (300 nM) and PhlTx1 (from 30 to 1000 nM) was efficient to almost completely eliminate the animal painful behavior induced by the injection of only OD1. Interestingly, no sign of cardiac or neuromuscular side-effects were detected during these experiments. This strongly suggests that, under our experimental conditions, the poor peptide selectivity towards the hNa_V_1.5 and 1.6 subtypes has no consequence in vivo. Anti-allodynic and analgesic effects of intrathecal injection of PhlTx1, without signs of neurotoxicity, were previously reported in a mouse model of acute and inflammatory neuropathic (paw formalin injection) pain [23,24]. However, intraplantar and intraperitoneal injections of the peptide were shown to be ineffective to produce analgesia in a Complete Freund’s Adjuvant (CFA)-induced heat hyperalgesia mouse model [25] and to affect mouse acute heat responses [26], respectively. It is likely that the discrepancy between these results is due to the route of PhlTx1 administration or the mouse model used, or both, rather than the dose of injected peptide. Further experiments are however needed to draw a definitive conclusion.

The effects of 9 toxins, showing a higher affinity than PhlTx1 for the hNa_V_1.7 subtype (with the following increasing order for mean IC_50_ values (between 0.9 and 130 nM): Pn3a, Df1a-NH_2_, m3-HwTx-IV, GpTx-1, ProTx-III, Cd1a, Df1a-OH, CcoTx2, and CcoTx1), were also tested in vivo on OD1-induced painful behavior of mice [14,18,20,26,29,30,32,39,41]. Although Pn3a has the highest affinity for the hNa_V_1.7 subtype (but see [24]), close doses of intraperitoneal injection of this toxin and intraplantar injection of PhlTx1 have similar effects on OD1-induced painful behavior of mice. In addition, a synergistic action between PhlTx1 or Pn3a and low, non-therapeutic doses of opioids (such as oxycodone or buprenorphine) or enkephalinase inhibitors (such as thiorphan) were recently shown to induce analgesia during the phase II of inflammatory pain in mice. From this point of view, PhlTx1 was reported to be more efficient than Pn3a (Patent # WO 2015036734 A1, 2015; [25,26]). This questions the existence of a correlation between a peptide hNa_V_1.7 affinity and its in vivo effects on rodent pain models. The evaluation of m3-HwTx-IV, GpTx-1, and ProTx-III, performed under similar experimental conditions as that of PhlTx1, revealed that these toxins produced a 10-, 5-, and 2-fold decrease of animal painful behavior induced by OD1, respectively. However, marked neuromuscular side-effects were reported for m3-HwTx-IV and GpTx-1. The other five toxins were also efficient to diminish or completely reverse the OD1-induced manifestations of pain but at relatively high concentrations (1 µM for Cd1a, CcoTx1, and CcoTx2, and 10 µM for Df1a-NH_2_ and Df1a-OH).

The relatively low affinity of PhlTx1 for the hNa_V_1.7 subtype motivated a SAR study. Our folding strategy provided reasonable yield of production. Its limitation appeared for some variants for which we were unable to obtain the desired peptide. In these cases, the directed folding strategy could be useful [24]. Among the eight peptide variant forms tested on HEK-293 cells overexpressing the hNa_V_1.7 subtype, W24A-, K25A-, and Y26A-PhlTx1 were shown to completely lose affinity for this type of channel subtype, indicating that the C-terminal residues Trp_24_, Lys_25_, and Tyr_26_ are essential for hNa_V_1.7 inhibition. It has already been mentioned in the literature that toxins belonging to the NaSpTx family 1 display positively charged amino acids Lys_25_, His_26_, Lys_27_, Lys_30_, surrounded by hydrophobic Phe_5_ and Trp_28_ clustered on one molecule face, which may be involved in the peptide interactions with TTX-sensitive Na_V_ channels [13,15,37,38]. However, PhlTx1 possesses only two of the four positively charged amino acids (K25 and R30 instead of K30), while the hydrophobic amino acids are almost preserved (Q5, instead of F5, and W28). This may explain the relatively low affinity of the peptide for the hNa_V_1.7 subtype, compared to the most potent toxins belonging to the NaSpTx family 1 which possess well-preserved H26 and K30, such as HnTx-III and HwTx-1 (IC_50_ < 200 nM and IC_50_ = 232 nM, respectively; see Table 2), as well as the well-known previously described antinociceptive toxins ProTx-III and GpTx-1 (IC_50_ = 2 nM for both, [14,40]). In addition, comparison of amino acid sequences between PhlTx1 and the 14 most similar or well-documented toxins described in the literature reveals that the three highly conserved residues Gly_4_, Pro_11_, and Trp_28_ are also crucial to govern hNa_V_ activity or to reinforce the hNa_V_1.7 inhibitory potency of ICK toxins [24,29,42,43]. This would have been further confirmed if correctly folded G4A-, P11A-, and W28A-PhlTx1 could have been obtained. On the other hand, the affinity of A1Z-, S8A-, K12A-, and K15A-PhlTx1 for hNa_V_1.7 was only weakly modified, which strongly suggests that the N-terminal group and the residues Ser_8_, Lys_12_, and Lys_15_ are not particularly important for the peptide hNa_V_1.7 activity. Finally, D7A-PhlTx1 was the only variant showing an improved affinity for hNa_V_1.7 (mean IC_50_ values of 47–73 nM for D7A-PhlTx1 vs. 254–309 nM for PhlTx1). However, a gain of affinity (between 2.5- and 7.4-fold) was also observed not only for the hNa_V_1.2 and 1.8 subtypes, but also for the hNa_V_1.5 and hCa_V_1.2 subtypes. 

## 4. Conclusions

The results obtained from the characterization of physicochemical and functional properties of PhlTx1 and some of its variants, on a wide range of ion channel subtypes, highlight the identification of a relatively interesting variant, D7A-PhlTx1, with potent analgesic effects in a mouse model of Na_V_1.7-mediated pain induced by OD1 but with a low improved affinity for the hNa_V_1.7 subtype and a modest degree of selectivity towards the other hNa_V_ subtypes.

## 5. Materials and Methods

### 5.1. Chemical Synthesis and In Vitro Folding of PhlTx1 and Its Variants

The assembly of different peptides was carried out using the stepwise solid-phase method with dicyclohexylcarbodiimide/1-hydroxy-7-azabenzotriazole (HOAT) as coupling reagents and N-methylpyrrolidone as solvent. Fmoc-protected amino acids were used with the following side chain protections: tert-butyl ester (Glu, Asp), tert-butyl ether (Ser, Thr, Tyr), trityl group (Cys, His, Asn, Gln), 2,2,5,7,8-pentamethyl-chromane-6-sulfonyl group (Arg), and tert-butyloxycarbonyl group (Trp). The peptides were assembled on a Fmoc-Phe(OtBu)-Wang resin (0.5 mmol/g loading), with the exception of the C-terminal-deleted (Delta-C-ter) variant which was assembled on a Fmoc-Cys(Boc)-Wang resin (0.55 mmol/g loading). Automated chain assembly was performed on a standard Applied Biosystems 433 peptide synthesizer (Applied Biosystems, Foster City, CA, USA). The different syntheses were run on a modified version of the Applied Biosystems standard 0.1-mmol small-scale program using 0.05 mmol of each resin. The linear peptide forms were purified from the crude synthetic products by analytical reversed-phase high pressure liquid chromatography (RP-HPLC). The maturation of peptides was performed in 100 mM tris-HCl buffer (pH 8) supplemented with 2 mM of reduced (GSH) and oxidized glutathione (GSSG), during 48 h at 4 °C. Mass spectrometry (MS) analysis was performed on a 4800 MALDI TOF/TOF™ Analyzer (Applied Biosystems).

Protected amino acid derivatives, resins, and dicyclohexylcarbodiimide were from Novabiochem (Meudon, France), HOAT from Applied Biosystems (Courtaboeuf, France), N-methylpyrrolidone from Carlo Erba Reactifs - Sds (Peypin, France), and GSH, GSSG, and L-cysteine from Sigma-Aldrich (Saint-Quentin-Fallavier, France).

### 5.2. Predicted 3D-Structure of PhlTx1

Computational modelling was performed, using the PyMOL software, to deduce the 3D-structure of PhlTx1 from those of closely related toxins archived in the protein data bank (PDB): ceratotoxin-1 (Cm1a, PDB: 6BRO), ceratotoxin-2 (Cm1b, PDB: 6BTV), F8A variant of Pre-1a (PDB: 5I1X), peptide Hs1a (PDB: 2MT7), hainantoxin-III (HnTx-III, PDB: 2JTB) and huwentoxin-I (HwTx-I, PDB: 1QK6).

### 5.3. Toxins and Chemicals

Lyophilized synthetic PhlTx1 wild-type and variant forms (purity rate > 97%) were dissolved in appropriate solutions to give adequate stock solutions. All compounds used as reference molecules to pharmacologically validate the current flowing through each channel subtype, as indicated in Table 3, were purchased from Sigma-Aldrich and Smartox Biotechnology (Saint-Egrève, France). 

The toxins were dissolved in distilled water to give a 100 µM stock solution, and the chemicals were dissolved in 100% dimethylsulfoxyde (DMSO, Sigma-Aldrich) to give a 10 mM stock solution. Just prior to experiments, successive dilutions were performed in the appropriate standard physiological medium (supplemented with 0.1% bovine serum albumin (BSA, Sigma-Aldrich) for toxins to avoid plastic adherence, and with 0.3% DMSO for chemicals to maintain solubility), to give the compound final concentrations indicated in the text.

### 5.4. Cell Lines Used for Functional Assays

Generation of inducible cell lines was achieved by cloning cDNAs encoding for hNa_V_1.2 (NM_021007.2) and hNa_V_1.5 (NM_000335) subtypes into the Flp-In® T-Rex® expression vector (Invitrogen, USA) and subsequently transfecting them into human embryonic kidney (HEK)-293 cell line, using the FuGENE® transfection reagent (Promega, France). The cell lines were cultured under standard conditions (37 °C, 5% CO_2_) in Dulbecco’s modified Eagle’s medium (DMEM) with GlutaMAX^TM^ supplement (Gibco, Thermo Fisher Scientific, Villebon-sur-Yvette, France), supplemented with 10% fetal calf serum (Invitrogen), 50 µg/mL hygromycin (Invitrogen), and 15 µg/mL blasticidin (Invivogen). They were sub-cultured every 3–4 days using accutase® (Sigma-Aldrich) or TrypLE Select (Gibco) enzyme. The cell line passage number was less than 15 on the day of experiments. Doxycycline (BD Biosciences, France) was added to induce target expression, at least between 12 and 24 h prior to experiments.

Recombinant HEK-293 cell lines, stably overexpressing the other hNa_V_ subtypes, were purchased from SB Drug Discovery (UK; hNa_V_1.3 and 1.4), ChanTest (Cleveland, OH, USA; hNa_V_1.6) and Eurofins (St Charles, MO, USA; hNa_V_1.1, 1.7 and 1.8/β1). These cell lines were cultured in Eagle’s minimum essential medium (MEM, Sigma-Aldrich; hNa_V_1.3 and 1.4), in DMEM/F-12 with GlutaMAX^TM^ supplement (Gibco; hNa_V_1.1, 1.6 and 1.8/β1) or in suspension in a FreeStyle^TM^ 293 expression medium (Gibco, hNa_V_1.7). The Chinese hamster ovary (CHO) cells heterologously overexpressing hCa_V_1.2/β2/α2δ1 (purchased from ChanTest) were cultured in Ham’s F12 nutrient mix with GlutaMAX^TM^ medium (Gibco), and those overexpressing hK_V_11.1 (purchased from B’SYS GmbH, Switzerland) in DMEM/F-12 nutrient mixture Ham’s medium (Sigma-Aldrich). All culture media contained 10% fetal bovine serum (Gibco) and selected antibiotics and additives, as recommended by the manufacturers. The cells were grown in flasks (37 °C, 5% CO_2_), and sub-cultured every 3–4 days using TrypLE Select enzyme. The cell line passage number was less than 20 on the day of experiments.

### 5.5. Automated Whole-Cell Patch-Clamp Electrophysiology

The effects of synthetic PhlTx1 and some of its variants were investigated on the cell lines overexpressing hNa_V_1.1–1.8, hCa_V_1.2, and hK_V_11.1 subtypes, using the automated QPatch HTX patch-clamp system (Sophion Bioscience, Denmark), allowing current recordings in whole-cell configuration and both signal acquisition and data analyses, as previously reported [15]. On the day of their use, HEK-293 and CHO cells were transferred into Eppendorf tubes containing a FreeStyle^TM^ 293 expression medium (Gibco) which were then placed in the automated electrophysiology platform. The extracellular medium composition for hNa_V_-overexpressing HEK-293 cells was (in mM): NaCl 154, KCl 4, CaCl_2_ 2, MgCl_2_ 1, and HEPES 10 (pH 7.4 adjusted with NaOH), and that of intracellular (i.e., path-clamp pipette) medium: CsF 150, EGTA/CsOH 1/50, HEPES 10, NaCl 10, MgCl_2_ 1, and CaCl_2_ 1 (pH 7.4 adjusted with CsOH). The extracellular medium composition for hCa_V_1.2-overexpressing CHO cells was (in mM): NaCl 145, KCl 4, CaCl_2_ 10, and HEPES 10 (pH 7.4 adjusted with NaOH), and that of intracellular medium: CsF 27, CsCl 112, EGTA 8.2, HEPES 10, NaCl 2, and Mg-ATP 4 (pH 7.4 adjusted with CsOH). The extracellular medium composition for hK_V_11.1-overexpressing CHO cells was (in mM): NaCl 145, KCl 4, CaCl_2_ 2, MgCl_2_ 1, HEPES 10, and D-glucose 10 (pH 7.4 adjusted with NaOH), and that of intracellular medium: KCl 120, CaCl_2_ 5.4, EGTA 10, HEPES 10, Mg-ATP 4, and MgCl_2_ 1.75 (pH 7.4 adjusted with KOH). Synthetic PhlTx1 and variants were diluted in the extracellular medium supplemented with bovine serum albumin (0.1%), to give the final concentrations indicated in the text. The time of cell incubation with peptides and reference molecules was adjusted to achieve steady-state effects, i.e., between approximately 2 and 7 min. The experiments were carried out at room temperature (20-22 °C).

In addition to the classical “closed-open” protocol, a “fully inactivated” protocol was also used to test an eventual inactivated-state-dependent effect of peptides on hNa_V_ subtypes. During the “closed-open” protocol, the hNa_V_-overexpressing HEK-293 cells were maintained at a holding potential of either −90 mV (hNa_V_1.5) or −100 mV (other hNa_V_ subtypes), and currents were elicited at a frequency of 0.2 Hz by 20 ms test-pulses to −20 mV (hNa_V_1.2 and 1.7), −40 mV (hNa_V_1.5) or 10 mV (hNa_V_1.8), preceded by 40 ms (hNa_V_1.7) or 200 ms (hNa_V_1.5) pulses to −120 mV, or not (hNa_V_1.2 and 1.8). The hCa_V_1.2-overexpressing CHO cells were maintained at a holding potential of −50 mV, and currents were elicited at a frequency of 0.05 Hz by 200 ms test-pulses to 0 mV. The hK_V_11.1-overexpressing CHO cells were maintained at a holding potential of −80 mV, and tail currents were elicited at a frequency of 0.07 Hz by 5 s test-pulses to −50 mV, preceded by 4.8 s pulses to 20 mV following 20 ms pulses to −50 mV. During the “fully inactivated” protocol, the hNa_V_-overexpressing HEK-293 cells were maintained at a holding potential of either −40 mV (hNa_V_1.1, 1.2 and 1.6) or −50 mV (other hNa_V_ subtypes), and currents were elicited at a frequency of 0.2 Hz by 20 ms test-pulses to −40 mV (hNa_V_1.5) or −20 mV (other hNa_V_ subtypes), preceded by 40 ms (hNa_V_1.3 and 1.4) or 20 ms (other hNa_V_ subtypes) pulses to −120 mV. The leakage current was not compensated for.

The concentration–response relationships were established by plotting the peak amplitude of the sum of ten cell currents recorded in the presence of a given molecule (Pm), expressed relative to that of the sum of these currents recorded before molecule application (Pc), against the molecule concentration ([m]). The theoretical concentration-response curves were calculated from typical sigmoid nonlinear regressions through data points according to the Hill equation (QPatch assay software): Pm/Pc = 1/[1 + ([m]/IC_50_) n_H_], where IC_50_ is the molecule concentration necessary to inhibit 50% of the response, and n_H_ is the Hill number.

### 5.6. Mouse Model of Na_V_1.7-Mediated Pain

The peptide analgesic effects were studied at room temperature in a mouse model of Na_V_1.7-mediated pain, based on intraplantar injection of toxin 1 from the *Odonthobuthus doriae* scorpion (OD1, Smartox Biotechnology) that potently enhances the activity of the Na_V_1.7 subtype by inhibiting channel fast inactivation and increasing peak current, as previously described [18]. The experiments were performed on 36 adult male C57BL/6 mice (*Mus musculus*, 8 weeks of age and 20–25 g of body weight), purchased from Charles River Laboratories (St Germain Nuelles, France). The animals were acclimatized for at least 48 h before experiments at the IGF animal facility where they were housed in a 12 h light/dark cycle and controlled temperature room, 3–4 per cage containing standard rodent chow and with ad libitum access to food and water. The experiments were conducted in compliance with the guidelines established by the French Council for animal care “Guide for the Care and Use of Laboratory Animals” (EEC86/609 Council Directive–Decree 2001-131), and the experimental protocols were approved by the French General Directorate for Research and Innovation (project APAFIS#747-2016012216345875v4 authorized to E. Bourinet) on 3 February 2019.

The painful behavior of mice was quantified by the number of spontaneous licking, flinching, shaking, and biting of the paw, for 10 min after intraplantar injection with 40 µL volume under light isoflurane anesthesia (AErrane®, Baxter S.A., Lessines, Belgique; 2% for 0.5–1 min), using a 50 μL micro-syringe. The injected solution comprised phosphate buffered saline (PBS) supplemented with 0.5% BSA and containing either 300 nM OD1 alone or OD1 and 30, 300 or 1000 nM of a given synthetic peptide. The study was experimentally designed, on the basis of previous experiments, to have six animals per group. The experiments were video recorded and blind analysis of mouse painful behavior was performed, off-line, by an observer unaware of which treatment each animal had received.

### 5.7. Statistical Analyses

Data are expressed as means ± standard deviations (S.D.) of either *n* independent experimental plates realized in triplicate or *n* mice. The statistical comparison of values was carried out using *(i)* the parametric two-tailed Student’s *t*-test (either paired samples for comparison within a single population or unpaired samples for comparison between two independent populations), or *(ii)* the one-way analysis of variance (ANOVA for comparison between the means of three or more independent populations) followed, if F was significant and if no variance inhomogeneity occurred, by post-hoc pairwise *t*-tests with Bonferroni correction. Differences between values were considered to be statistically significant at *P* ≤ 0.05.

## Figures and Tables

**Table 1:** Production as well as biochemical and pharmacological characterizations of wild-type (WT) PhlTx1 and its variants obtained by an alanine-substituted approach.

PhlTx1	Production			Biochemical Characterization		Pharmacological Characterization ^1^
	Linear (mg)	Oxidized (mg)	Folding yield (%)	Theo. Mass	Exp. Mass	hNa_V_1.7 IC_50_ (nM)
WT	150	17.3	12	4055.72	4055.73	254.3 ± 147.6 (5)
A1Z *	9.8	1.1	11	4095.71	4095.71	171.5 ± 51.0 (5)
D7A	12.1	2.5	21	4011.73	4011.72	47.0 ± 40.9 (3)
S8A	9.9	0.55	6	4039.72	4039.70	179.5 ± 118.4 (4)
K12A	9.5	0.75	8	3998.66	3998.67	252.5 ± 13.6 (4)
K15A	8.6	1.1	13	3998.66	3998.66	706.4 ± 87.8 (5)
W24A	9.0	1.1	12	3940.68	3940.68	> 10000 (4)
K25A	9.2	2.0	22	3998.66	3998.67	> 10000 (3)
Y26A	11.1	2.5	23	3963.69	3963.68	> 10000 (6)

^1^ Mean ± S.D. of data obtained from n different plates (number in parentheses) realized in triplicate, obtained by using the “fully inactivated” protocol; * Z: Pyroglutamic acid; Theo. Mass: theoretical mass; Exp. Mass: experimental mass.

**Table 2:** Comparison of amino acid sequences between PhlTx1 and the 14 most similar or well-documented toxins described in the literature. The sequence alignment was performed with ClustalIW (version 12.1 from Emboss programs, EBlosum62 matrix for two pair alignment). The cysteine scaffold and conserved residues are indicated in blue and red, respectively. The Ala-substituted amino acids producing a complete loss of variant affinity for the hNa_V_1.7 subtype are colored red (W24A, K25A, and Y26A), those resulting in a less than 2.8-fold change variant affinity are colored orange (A1Z, S8A, K12A, and K15A), and the one (D7A) resulting in a gain of variant affinity is colored green (see also Table 1). The percentage of sequence identity with PhlTx1, the known targets, and the mean IC_50_ value (in nM) reported for the hNa_V_1.7 subtype are described in the right-hand columns, according to data sourced from Pubmed and ArachnoServer [31].

Name *		Amino Acid Sequence	Identity	Target	IC_50_/hNa_V_1.7 (nM)
PhlTx1	(µ-TRTX-Pspp-1)	--A--CLGQWDSCDPKASKCCPNYACEWKYPWCRYKLF-	100%	Na_V_	254
OAIP2		--D--CLGQWASCEPKNSKCCPNYACTWKYPWCRYRAGK	76%	?	-
TlTx1	(κ-TRTX-Tb1a)	-AA--CLGMFESCDPNNDKCCPNRECNRKHKWCKYKLW-	59%	K_V_	-
Osp1b	(µ-TRTX-Osp1b)	--E--CLGWMKGCEPKNNKCCSSYVCTYKYPWCRYDL--	58%	Na_V_	-^1^
TlTx2	(κ-TRTX-Tb1b)	-DD--CLGMFSSCDPKNDKCCPNRVCRSRDQWCKYKLW-	56%	K_V_	-
Cd1a	(β-TRTX-Cd1a)	--D--CLGWFKSCDPKNDKCCKNYSCSRRDRWCKYDL--	55%	Na_V_	16 [30]
CcoTx1	(β-TRTX-Cm1a)	--D--CLGWFKSCDPKNDKCCKNYTCSRRDRWCKYDL--	55%	Na_V_	2-130 [29,32]
CcoTx2	(β-TRTX-Cm1b)	--D--CLGWFKSCDPKNDKCCKNYTCSRRDRWCKYYL--	55%	Na_V_	95 [32]
TlTx3	(κ-TRTX-Tb1c)	-DD--CLGMFSSCDPNNDKCCPNRVCRVRDQWCKYKLW-	53%	K_V_	-
Pre1a	(β/δ-TRTX-Pre1a)	-ED--CLGWFSRCSPKNDKCCPNYKCSSKDLWCKYKIW-	53%	Na_V_	114 [33]
Hs1a		GND--CLGFWSACNPKNDKCCANLVCSSKHKWCKGKL-	52%	Na_V_	- [34]
HnTx-III	(µ-TRTX-Hhn2a)	----GCKGFGDSCTPGKNECCPNYACSSKHKWCKVYLGK	50%	Na_V_	232 [28]
Ccy1b	(µ-TRTX-Ccy1b)	-DD--CLGFFKSCNPDNDKCCENYKCNRRDKWCKYVL--	48%	Na_V_	- [13]
Pmr1a	(µ-TRTX-Pmr1a)	-DD--CLGMFSSCDPDNDKCCEGRKCNRKDKWCKYVL--	48%	Na_V_	- [35]
HwTx-I	(µ/ω-TRTX-Hs1a)	----ACKGVFDACTPGKNECCPNRVCSDKHKWCKWKL--	45%	Na_V_/Ca_V_	<200 [36]

* The Greek letter(s) before the toxin name is associated to its type of action: µ for Na_V_ channel inhibition, β for shift in the voltage-dependence of Na_V_ channel activation, δ for delay inactivation of Na_V_ channels, ω for Ca_V_ channel inhibition, and κ for K_V_ channel inhibition. TRTX, theraphotoxin.

**Table 3:** Compounds used as reference molecules to pharmacologically validate the current flowing through each hNa_V_, hCa_V_, and hK_V_ channel subtype.

Channel Subtype	Drug Reference				
	Name	Molecular Mass	Purity Rate	Provider	IC_90–95_^1^ (µM)
hNa_V_1.1–1.8	Tetrodotoxin (citrate)	319.27	> 98%	Sigma-Aldrich	1
hCa_V_1.2	Nifedipine	346.33	> 98%	Sigma-Aldrich	10
	Cadmium (chloride) ^1^	183.32	> 99%	Sigma-Aldrich	200
hK_V_11.1	BeKm-1	4091.70	> 97%	Smartox Biotechnology	1

^1^ Compound concentration necessary to inhibit 90–95% of current.
